# Robot-Guided Evacuation as a Paradigm for Human-Robot Interaction Research

**DOI:** 10.3389/frobt.2021.701938

**Published:** 2021-07-15

**Authors:** Alan R. Wagner

**Affiliations:** Robot Ethics and Aerial Vehicles Laboratory, Department of Aerospace Engineering, The Pennsylvania State University, University Park, PA, United States

**Keywords:** evacuation, human-robot interaction, robot ethics, emergency robotics, human-robot trust

## Abstract

This paper conceptualizes the problem of emergency evacuation as a paradigm for investigating human-robot interaction. We argue that emergency evacuation offers unique and important perspectives on human-robot interaction while also demanding close attention to the ethical ramifications of the technologies developed. We present a series of approaches for developing emergency evacuation robots and detail several essential design considerations. This paper concludes with a discussion of the ethical implications of emergency evacuation robots and a roadmap for their development, implementation, and evaluation.

## Introduction

The field of Human-Robot Interaction (HRI) tends to focus on service, education, entertainment, and healthcare applications (Bartneck, et al., 2020). HRI applications in these areas lend themselves to laboratory development and eventual experimentation in real-world settings. Moreover, for the most part, the human that is the focus of the service, educational experience, being entertained or whose health is being attended to, is typically relaxed, affable, attentive to the robot, and contemplative of the robot’s performance on its assigned tasks.

The psychological disposition and resulting behavior of people using a robot for these applications stands in stark contrast to the use of robots for search and rescue or emergency evacuation applications. During search and rescue or emergency evacuation people tend to be emotional, tense, confused, inattentive, pliable, and reactive to the robot without consideration of its performance. In other words, rescue and emergency evacuation situations tend to put people in a different state of mind than traditional HRI application areas. Although, on the surface it may appear as if a dichotomy exists between applications in these two areas, in reality people’s behavior can differ from day-to-day. While being served, taught, entertained, or treated, occasionally people will be emotional, tense, and reactive. It therefore behooves the HRI community to consider and explore both sides of the human state of mind in order to develop robots that might be capable of prolonged interaction with people and responsive to their daily psychological states.

With this in mind, our research examines the development and use of mobile robots as guides leading human evacuees to safety. We focus on the evacuation of buildings that contain large numbers of people, high-rise residential complexes, schools, and shopping malls, for example, because we believe that emergency guidance robots placed in these buildings could save a significant number of lives. At least with respect to high-rise residential complexes, the global number of these buildings is increasing (CTBUH, 2018) and the evacuation of these buildings is a complex and time-consuming process (Gershon et al., 2007). For example, after the 1993 World Trade Center bombing people could be found at their desk 6 h after the beginning of the evacuation (Fahy RF, 1995). Many high-rise buildings are not designed for rapid emergency evacuation (Meacham, 1999). Moreover, rescue of occupants by first responders is dangerous and takes a long time after the beginning of the emergency (Gershon et al., 2012).

**FIGURE 1:**
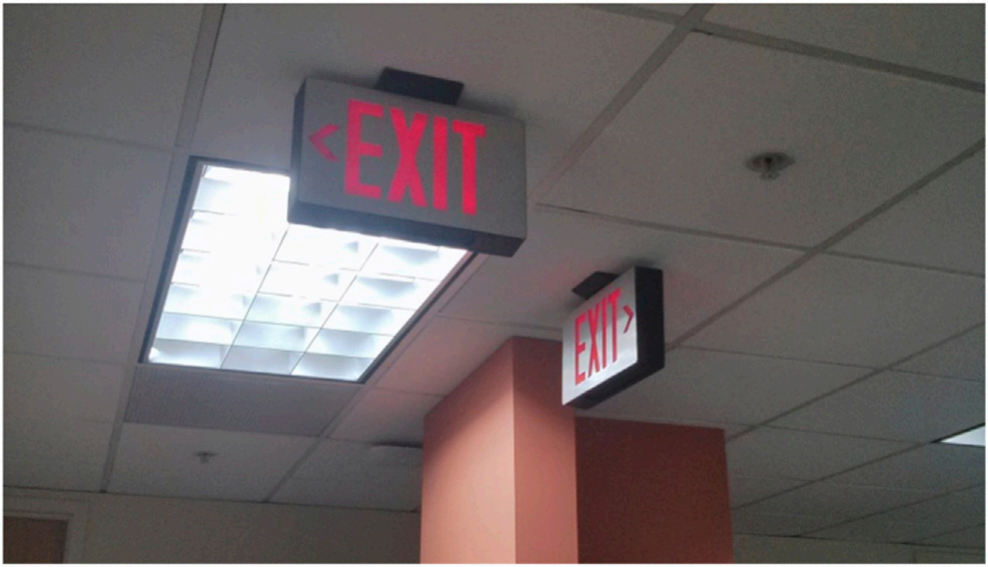
Confusing exit signs.

In its broadest formulation, robot-guided emergency evacuation tasks the robot with leading individuals or groups of people away from danger to a safe location. This broad formulation can, however, be delimited in a variety of different ways to make the problem more tractable without necessarily compromising real-world applicability. For example, if the robot is tasked with evacuating residents from an apartment building, then the robot can be provided with a priori information about the location of exits, stairwells, and potential evacuees. Moreover, many evacuation environments have ample infrastructure, such as WIFI, to make the task easier. These and other reasonably grounded assumptions simplify the robot-guided emergency evacuation task. Traditional, non-robotic approaches to emergency evacuation include the use of exit signs, flooring lights, and broadcast announcements. These approaches are reasonably effective as a means of communicating a pathway to an exit. But traditional approaches to emergency evacuation are static and may not be well informed about the emergency. For example, during the 2001 World Trade Center bombing announcements over the public address systems told evacuees to return to their desks (Averill et al., 2013). Exit signs can be confusing (Figure 1) and some exits may be blocked or overcrowded. Robot emergency evacuation guides may therefore be able to adapt to the emergency in real time to save lives.

As with many HRI problems, robot-evacuee interaction is dictated by the context, the evacuee(s), and the robot. Contextual factors include the cause and type of emergency, the location of the evacuation, and the ease of exiting. Evacuation from a school, for example, is physically less demanding than evacuation from a high-rise building because a large number of stairs must traversed in order exit a high-rise building. The type of evacuee includes factors such as the person’s age, disabilities, or psychological state. For instance, evacuating children may require different methods and styles of communication than evacuating adults. Health and especially health limitations can affect one’s ability to evacuate and understand the robot’s directions. Finally, factors related to the robot include how the robot should be designed, the modalities it uses to communicate with evacuees, and how it uses its communicative behaviors and mobility to successfully and quickly evacuate people.

The remainder of this paper begins by presenting a rationale for robot-guided evacuation. Next we describe different approaches to robot-guided evacuation, including a discussion of how to formulate the problem of robot-guided evacuation. We then discuss principles for robot design followed by an examination of the ethical implications of robot-assisted evacuation. We conclude by presenting a roadmap for this application domain.

## Why Evacuation?

There are a number of reasons that robot-guided emergency evacuation is a valuable human-robot interaction problem. First, and perhaps most importantly, robots might one day be developed to serve as an instantaneous first responders immediately reacting to an emergency by contacting the authorities and guiding people to safety. During an emergency, people are often initially confused (Proulx, 2003). Information and leadership, even in the form of a robot, may be important for initiating the evacuation process (Bryan, 2002). Ideally, these robots will save lives by reducing the time required for evacuation, increasing the number of people evacuated, reducing crowding at exits, and providing timely information about the emergency to evacuees.

Evacuation robots might not only protect the lives of evacuees but also reduce the risks for first responders. By providing information about the emergency to first responders the robot might be able to alleviate some of the risks to first responders. For instance, simply providing camera images or streaming video could help first responders gauge the nature of the situation. Moreover, robots might be developed that could be remotely controlled, thereby allowing first responders to intentionally gather information about the emergency or how evacuees are responding to the emergency. One can imagine an advanced 911 operator that responds to emergencies in apartment buildings or schools by taking control of an onsite robot to provide additional up-to-date information for police officers and fire fighters.

Robot-guided evacuation also allows HRI researchers to investigate how humans in a highly aroused and potentially emotional state of mind interact with a robot. The vast majority of HRI research focuses on applications developed for staid, controlled environments such as one’s home or the classroom (Kidd and Breazeal, 2007; Park et al., 2017; Zachiotis et al., 2018). Very little HRI research has examined situations in which the human or humans are under pressure or threat of physical harm. People act differently during an emergency (Klein et al., 1986; Jansen et al., 1995). Interacting with a person that is fleeing from some threat is, in many ways, fundamentally different from interacting with a person in a laboratory environment. Fight-or-flight responses can be debilitating and impair judgment. Evacuee-robot interaction may therefore demand a unique perspective on how a robot should behave. An evacuation robot may need to adjust its behavior based on the person’s reaction and emotional state; it may need to be authoritative and interact with a commanding presence in order to convince people when and how to leave (Kuligowski, 2008; Robinette et al., 2012). The dynamic nature of the evacuee-robot relationship presents challenges as well as opportunities for important and novel research.

For many HRI applications, ecological validity is simply assumed (Dragan et al., 2013; Bartneck, et al., 2020; Chen et al., 2018). It may be expected that HRI research performed in a well-controlled laboratory experiment will extrapolate to more realistic settings. For some applications, such as service robots, such assumptions may be warranted (King et al., 2010; Mast, et al., 2015). For applications such as emergency evacuation, on the other hand, researchers cannot assume that results and data gathered from laboratory experiments will inform how real people react to a real emergency. Because externally invalid simplifications could eventually increase the risks to evacuees, we argue that research in this area must include real-world experiments with real robots operating as they would during a real emergency. Although challenging, these experiments serve to moor simulation experiments and well-controlled laboratory experiments to reality. These real-world experiments may allow a researcher to compare the results from simulation experiments to results from laboratory experiments to results from real-world experiments in a way that few other applications allow. This is not to say that simulation experiments do not have a role to play in this type of research. We are merely arguing that the results from simulation experiments should be supported by real-world experiments.

Finally, emergency evacuation can be used as a domain to study a variety of important HRI problems. For example, evacuation can be used as means for gauging the efficacy of an explanation (Nayyar et al., 2020), estimating a person’s emotional state, or quantifying the impact of trust repair methods (Robinette et al., 2015). As a paradigm, emergency evacuation lends insight to exploring both how to develop interactive robots and how people respond to robots.

The section that follows reviews the robot-guided evacuation research.

## Review of Related Work

There has been substantial work on the mathematical modeling of large-scale evacuations of an urban populace (Verdiere, et al., 2014; Song et al., 2014; Song, et al., 2017). However, robot-guided evacuation has only very recently been studied (Robinette et al., 2014; Boukas et al., 2015; Robinette et al., 2016a). For example, (Boukas et al., 2015) use cellular automata to model crowd dynamics and test the system by having a robot guide human subjects during a simulated evacuation showing that their robot can improve evacuation times and influence approximately 12% of the evacuees to follow the robot’s guidance. Outside of our research this is the only example of an evaluation of a physical robot in a human subject evacuation experiment. Other related work has examined the several related challenges associated with robot-guided evacuation. For example, (Jiang et al., 2016) employed robots as dynamic obstacles near exits to improve the evacuation efficiency using a social force model. The existing work clearly demonstrates that robots are able to speed the evacuation process.

It is worth pointing out that the aforementioned research only considers single robots. Robot-guided evacuation involving multiple robots is quite limited. A cooperative exit-seeking algorithm for robots is designed in (Zhang and Guo, 2015) to guide evacuees using online estimation of the gradient and tracing gradient-descent while maintaining a predefined formation in movement. A similar idea is implemented by (Tang et al., 2016) where an algorithm was developed to help pedestrians find the best exit with the shortest escape time. However, current multi-robot evacuation systems are only validated in simulation and lack detailed coordinated motion planning strategies and human-robot interaction studies (Sakour and Hu, 2017). We are thus motivated to develop systematic methods of designing coordinated robot decision-making and motion planning in human crowded environments to achieve an efficient evacuation, investigate the human-robot interaction issues associated with evacuation through real human-robotic experimental studies, and evaluate the effectiveness of our theoretical and experimental results by creating a coordinated multi-robot evacuation system and field testing these systems.

The next sections attempt to organize the various aspects of the robot-guided emergency evacuation problem. This section also seeks to codify the goals and metrics for success of different approaches to this problem.

## Approaches to Robot-Guided Emergency Evacuation

There are different approaches to robot-guided emergency evacuation that can be taken depending on characteristics of the robot, such as its ability to autonomously move around the environment, and the number of robots available. In general, we assume that a non-trivial amount of tuning to the environment is necessary and will be completed prior to deployment of the evacuation robots. Typically a map and the location of the building’s exits will be necessary. Information about irregular flooring or visual codes placed into the environment itself may also be required. Moreover, large alterations to the map, such as hallway or exit closures will also present problems. In the worst case the robot could guide evacuees to an exit that no longer exists. Just as other types of emergency equipment requires periodic (often annual) updates and testing, we believe that emergency evacuation robots will also require annual testing.

The accumulation of clutter in the environment can present navigation and perception problems for the robot and the evacuees. Such clutter may represent a hazard irrespective of the use of emergency robots. Only if the evacuation robots become stuck in or part of the clutter itself does the use of emergency robots add to this risk. To prevent choke points, clutter should not be allowed to accumulate in buildings that may require evacuation.

The sections below describe some approaches the have been explored by our lab. It is important to note that these approaches range in the technical complexity needed for the robot to operate as a guide.

### Actuated Traffic Cop

**FIGURE 2:**
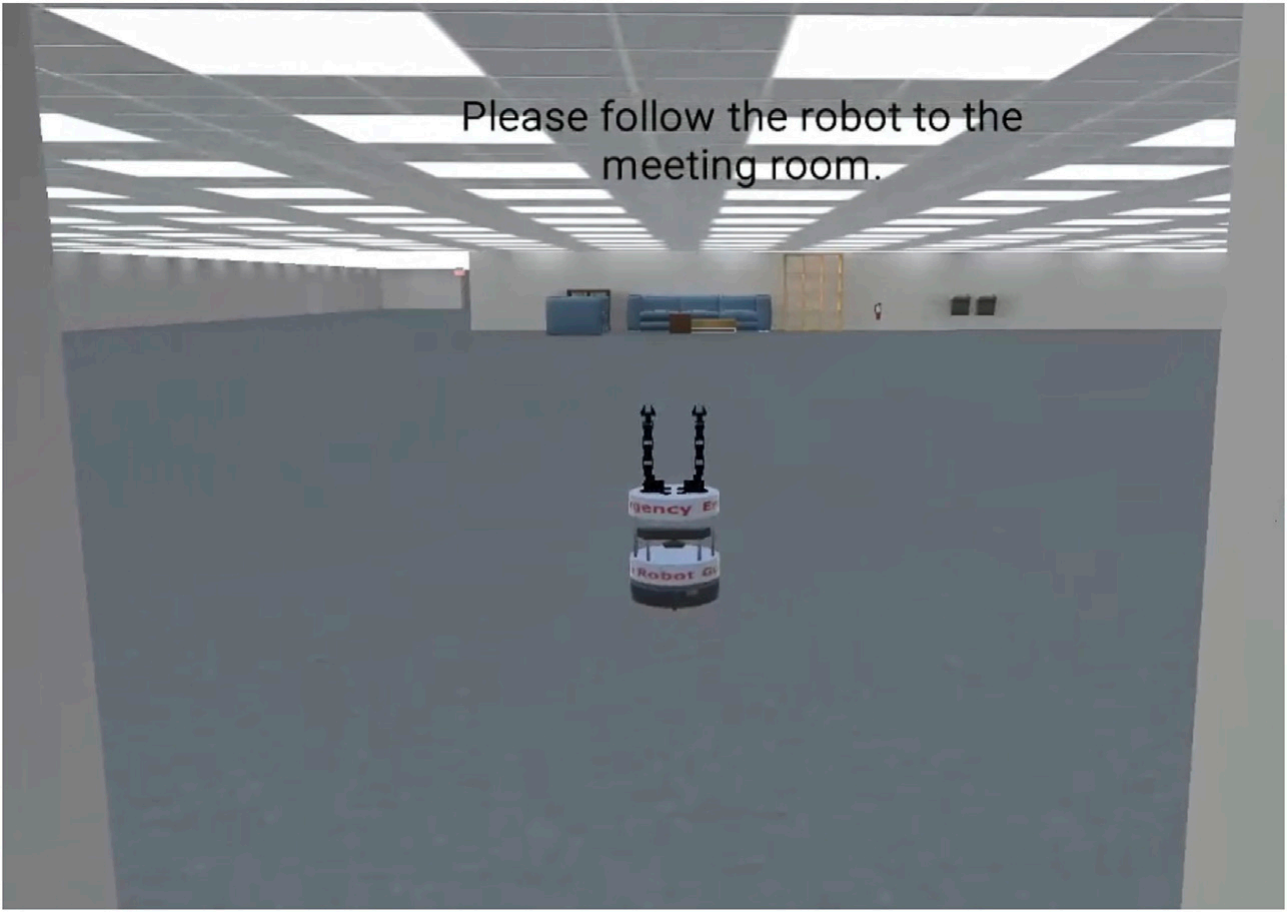
This image depicts an emergency evacuation robot meeting a human subject at the entrance to an office building. The robot leads the subject to a meeting room in the environment.

A less complex, yet still robotic, approach to developing an emergency evacuation robot is to simply create a robot capable of moving to a fixed, known and nearby location in the evacuation environment to act as a type of automated traffic cop during an emergency evacuation. When an emergency occurs, these robots are activated to move to a predefined location, such as a corridor, to direct evacuees toward an exit (Figure 2). The robot may have limited or no ability to interact with humans. Alternatively, the robot may be able to broadcast verbal or visual messages but incapable of responding to inquiries.

Although limited in its interactive capabilities, actuated evacuation traffic cop robots may nevertheless improve evacuation by directing people away from crowded exits or providing situation awareness for first responders. These robots may even be programmed to count the number of evacuees to generate a rough estimate of the number of people still in the building. From a practical perspective, traffic cop style robots present the least technically challenging form of evacuation robot. Moreover, this style of evacuation robot has the potential to evolve into more technically complex and nuanced versions with time and research. As such it represents more of a starting point than an end goal.

### Multi-Robot Handoffs

**FIGURE 3:**
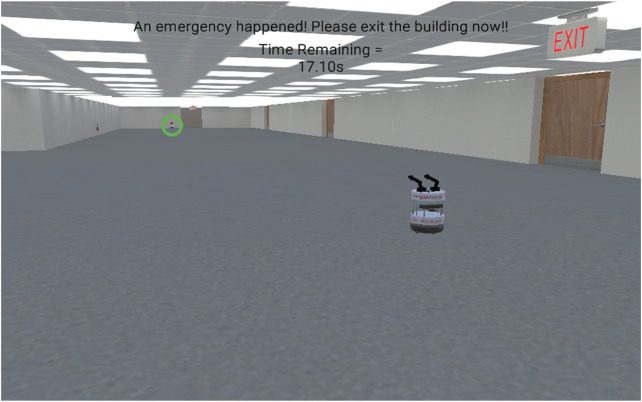
An example of the use of multi-robot handoffs. The nearby robot does not move. It simply directs the subject to the far away robot within the green circle.

**FIGURE 4:**
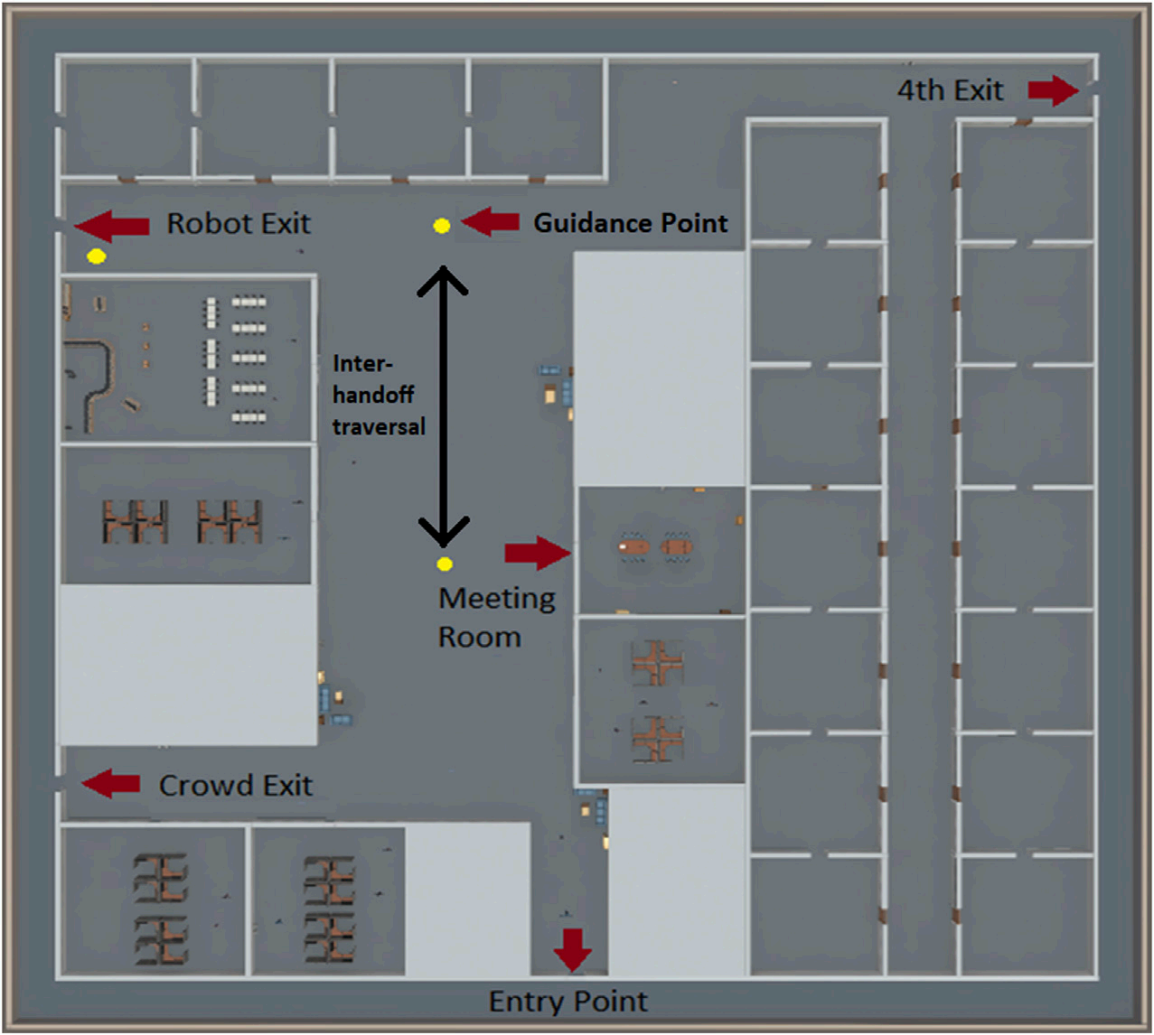
An overhead map of our emergency evacuation environment. The red arrows highlight several key locations used for our emergency evacuation experiments. The yellow circles depict guidance points. The black arrow depicts an inter-handoff traversal.

A multi-robot version of the actuated traffic cop approach described above allows for more nuanced guidance of groups and crowds by serially directing evacuees from one robot to the next (Figure 3), essentially handing off the guidance responsibility from one robot to the next. For this approach, when an emergency occurs several individual actuated traffic cop robots move to predetermined evacuation guidance points, for example multi-junction corridors. Guidance points are points where the evacuee needs to make a decision about which direction to go. These points are typically corridor intersections or places where a corridor branches. At a set of predefined guidance points, each robot uses arm motions and verbal statements to encourage evacuees to move in a specific direction. Evacuees follow the robot’s guidance moving in the specified direction until either the evacuees encounter another robot or they arrive at the exit. We denote the path from one robot to the next robot the inter-handoff traversal. The robots coordinate their guidance directions to funnel evacuees toward the safest nearby exit. Figure 4 depicts an overhead map of a multi-robot handoff evacuation depicting these concepts in a simulation of an office building. For example, in a school evacuation students from a classroom may encounter the first robot outside the door of their classroom. This robot directs them to a four-way hallway intersection where they encounter another robot directing them down the corridor to the rear of the school. At the end of the corridor they encounter a final robot directing them to an exit at the end of a hallway. Hence, the evacuee is handed off from one guidance robot to another guidance robot until arriving at a safe exit.

Although each individual robot is reasonably simple in its perceptual, decision-making, and behavioral capabilities, having a multi-robot evacuation team significantly increases the complexity of the system when compared to a single actuated traffic cop. Nevertheless, this cost comes with the benefit of increasing the system’s ability to guide evacuees to possibly distant exits in order to avoid crowding or other dangers associated with a nearby exit. The system may also be more robust because the presence of multiple robots increases the likelihood that an evacuee will notice guidance directions of one robot even if a nearby robot has failed. Moreover, multi-robot handoffs can still provide situation awareness by observing evacuees as they pass by or the environment from a fixed direction. This system can redirect evacuees to a different exit if the nature of the emergency changes, but evacuees may become confused or disoriented by the change in directions, especially if they are currently moving between two robots and one robot directs them to go back in the direction from which they came. Finally, the robot’s limited mobility (i.e., inability to climb stairs or lack of speed) will not impact its ability to provide emergency evacuation directions.

On the other hand, robot handoffs do limit the guidance that the robot can provide. Specifically, the robot may not be able to adapt to issues that arise while the person is traveling from one robot to the next. Additionally, evacuees may not be able to see the robot that they are moving toward, leading to confusion and possibly slowing their evacuation (Figure 3). Moreover, multi-robot handoff systems may be better suited for some environments and some emergencies than others. For example, open area environments such as sporting events may increase the visibility of the next robot whereas hotels with short, winding hallways may limit the evacuee’s visibility of the next robot. Further, multi-robot handoffs may also be well suited for earthquakes because the robot does not need to travel far to reach its guidance point, whereas handoffs may be less well-suited for fires again because of limited visibility.

### Shepherding

**FIGURE 5:**
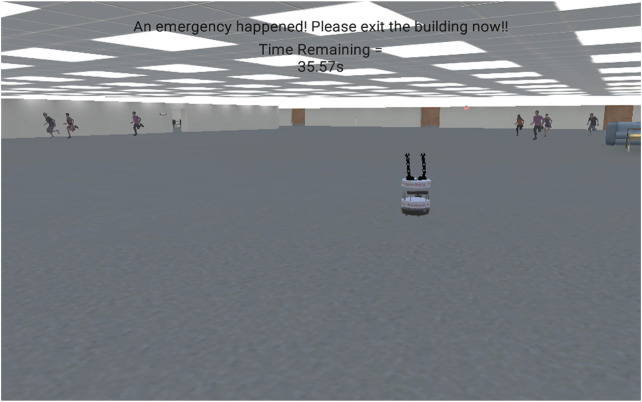
A sheperding robot guiding an evacuee to an exit. The robot travels in front of the evacuee all of the way to the exit.

In contrast to multi-robot handoffs, shepherding is an approach to robot-guided evacuation in which the robot leads individual evacuees or groups of evacuees to an exit (Figure 5). In this case, when an emergency occurs the robot may move to a specific location where evacuees may be known to congregate, or simply search for evacuees to help. Upon locating potential evacuees the robot engages the evacuees either asking if they need help finding an exit or, in some situations, authoritatively demanding that the evacuees follow the robot to an exit. The robot then leads, or shepherds, the evacuees to an exit, before returning to another congregation point.

One advantage of shepherding is that the robot remains near the evacuee(s) at all times. This may allow the robot to provide the evacuees with information or observe any medical issues that occur. Shepherding also allows the robot to tailor its behavior to the evacuee(s). For instance the robot can reduce its speed to match the speed of the evacuee. Shepherding also allows the robot to dynamically alter its evacuation path as dictated by the situation and explain to the following evacuees why such a change was necessary. Moreover, simulation experiments that have compared the shepherding approach to the handoff approach for robot-guided evacuation have found that shepherding results in a greater decrease in evacuation time (Nayyar and Wagner, 2019).

The major disadvantage of shepherding is the technical complexity necessary to develop and test a reliable system. Creating an autonomous shepherding robot that operates during an emergency is technically challenging. Even if the robot possess a great deal of prior knowledge about the building, including a floor plan, the location of exits, and accurate localization information, the robot will still need to navigate around obstacles, move quickly, recognize evacuees and determine if the evacuees are following or ahead of the robot. Because of these challenges, to the best of our knowledge shepherding robots have only been developed for simulation environments.

In a recent virtual experiment we compared a human participant’s decision to follow the robot during an emergency when the robot evacuation approach was multi-robot handoffs vs. shepherding (Nayyar and Wagner, 2019). In this experiment, remote participants are guided to a meeting room by a robot that either made mistakes or made no mistakes. While in the room performing a nominal task an emergency occurs. The robot offers to guide the evacuee to an exit to the right using one of the two approaches while either witnessing or not witnessing a crowd of individuals running to the left. In general we found that the shepherding approach convinces significantly more evacuees to follow the robot. When there is no crowd fleeing in the opposite direction and the robot had not previously made a mistake, 75% of subjects followed the robot when it shepherded them to an exit. The percentage following the robot drops to 60% if the robot had previously made a mistake, 45% if there is a crowd fleeing in the opposite direction but no prior robot mistake, and less than 12% if the subject witnesses a crowd fleeing and the robot has recently made a mistake. When handoffs are used the percentage of subjects that follow the robot drops to 27% (no mistake, no crowd), 19% (mistake, no crowd), 2% (no mistake, crowd), and 3% (mistake, crowd), respectively. These results suggest that evacuees will be more likely to follow a robot that shepherds. Still, significant technological advances will be needed for shepherding to be possible during an emergency.

### Multi-Robot Shepherding and Handoffs

The most complex approach to robot-guided emergency evacuation is one that combines multi-robot shepherding with handoffs. For this approach, multi-robots react to changes in the situation by switching between handoffs and shepherding as needed. For example, this approach could operate by initially taking a handoff approach until a large number of evacuees have exited and then, once the majority of the building is empty, patrol the building seeking to identify stragglers and shepherding these stragglers to a nearby exit. This approach may also be necessary when individuals are hiding or too frightened to move to an exit without an escort. Although technically challenging, this approach follows naturally once a system that is capable of shepherding has been developed.

### Shelter in Place Situations

Some types of emergencies, such as active shooter situations or tornados, require that people shelter in place. For these types of situations an evacuation robot can still be useful. During active shooter situations the robot can simply patrol hallways broadcasting information such as warnings that there is currently an active shooter on the premise and that all people should shelter in place. The robot can also broadcast updates about the situation as it changes. Likewise, information about an incoming tornado can keep people abreast of changes in the situation and when it is safe to evacuate. To the best of our knowledge robot assistance during a shelter in place situation has not been investigated by researchers. A summary of the advantages and disadvantages of each approach is provided in Table 1.

**TABLE 1:** Summary of different robot-guided evacuation approach advantages and disadvantages.

Approach Name	Approach advantages	Approach disadvantages
Actuated traffic cop	Relatively simplistic implementation in ecologically valid environments. Approximate technology readiness level (TRL) 5–6. Appropriate for a wide range of different emergencies	Limited ability to respond to dynamic emergency situations
Multi-robot handoffs	Moderately difficult implementation in ecologically valid environments. Approximate technology readiness level (TRL) 4–5. Capable of dynamically redirecting to different exits. Multi-robot system may increase robustness	Guidance directions conveyed over a distance and are not personalized or adapted to the evacuees. Multi-robot system adds complexity
Shepherding	Capable of dynamically redirecting to different exits. Guidance directions can be personalized to the evacuee or evacuee group	Complex implementation in ecologically valid environments. Approximate technology readiness level (TRL) 3
Multi-robot shepherding and handoffs	Capable of dynamically redirecting to different exits. Guidance directions can be personalized to the evacuee or evacuee group. Capable of dynamically redirecting to different exits. Multi-robot system may increase robustness	Complex implementation in ecologically valid environments. Approximate technology readiness level (TRL) 3
Shelter in place situations	Relatively simplistic implementation. Near-term technology readiness	Appropriate only for certain types of emergencies

## Robot and Experimental Design for Robot-Guided Evacuation

### Robot Design

Our previous work has shown that *in-situ* robots can improve existing technology, such as static emergency exit signs and alarms, by communicating the conditions of the emergency site to command posts while finding and guiding victims of the emergency out of danger (Robinette and Howard, 2011; Robinette and Howard, 2012). Conveying guidance information to a small percentage of evacuees can dramatically improve survivability (Robinette et al., 2012).

**FIGURE 6:**
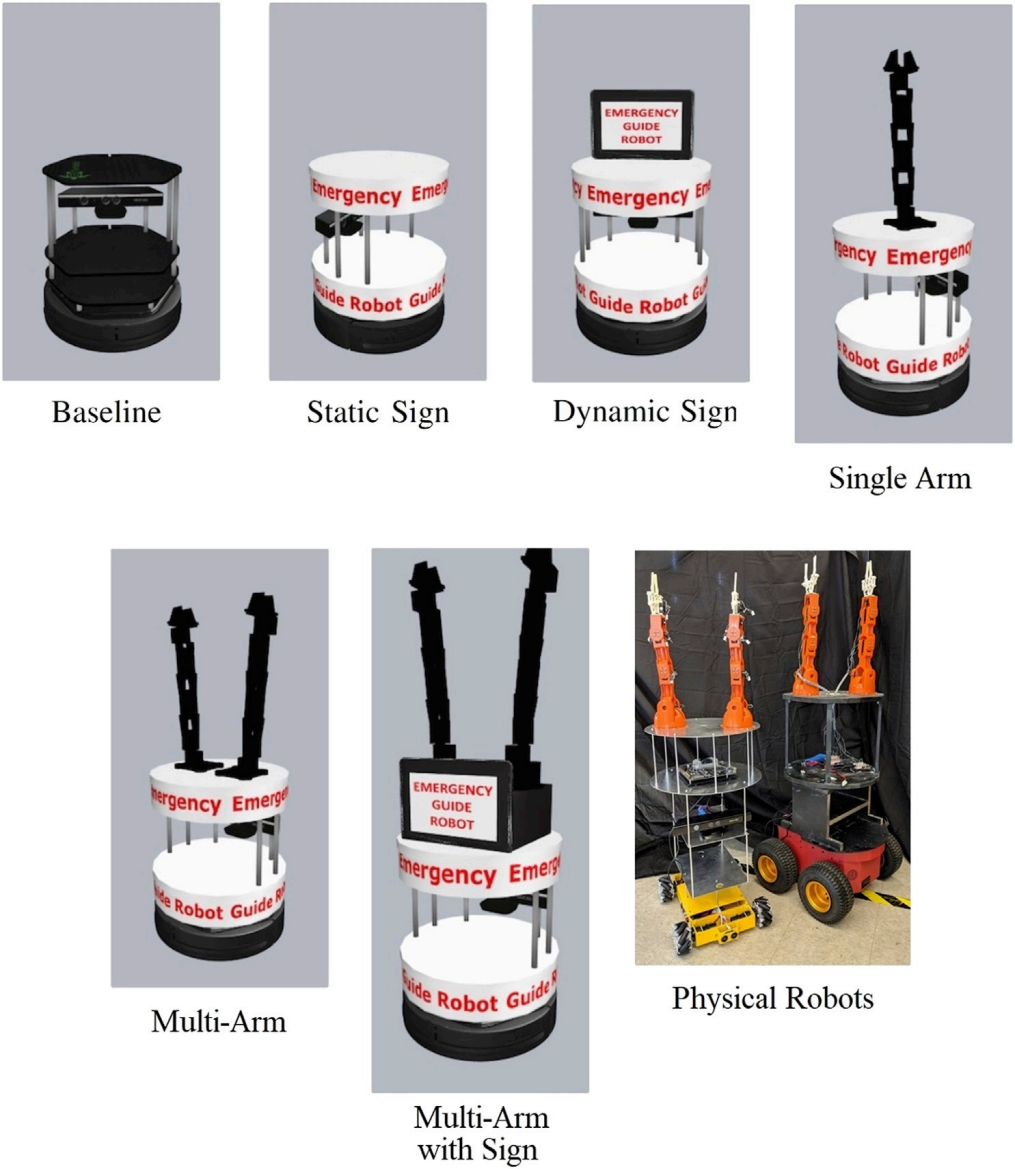
Different robot designs evaluated for its ability to communicate guidance directions. The physical robots are based on the multi-arm design.

Our prior work in this area examined how best to construct a mobile robot that could convey understandable directions to evacuees (Robinette et al., 2014). Figure 6 depicts several of the different robot designs tested. We considered three categories of visual methods for conveying guidance information: static signs, dynamic signs, and arm gestures. We combined these categories with each other and a mobile robot base to form five different platforms with information conveyance packages and one baseline platform with no specialized information conveyance abilities. The information conveyance ability of these robots was tested by recording simulations of the six resulting platforms performing each of four guidance instructions at both an instruction point near an evacuee and a point further away from the evacuee. Human participants then interpreted the instructions and rated the understandability of the information being conveyed. Our results showed that a ground platform with a dynamic display and multi-arm gestures provides the clearest instructions to evacuees during an emergency. We also found that adding seemingly trivial aesthetics such as signs can produce differences in outcomes of human-robot interaction experiments.

A follow-up experiment was conducted examining the difference between virtual vs. remote vs. physical robots and environments (Robinette et al., 2016b). The remote presence experiment tasked human subjects with watching a video of a physical robot attempting to convey directions at close and far distances. The physical experiment repeated the remote experiment with in person subjects and a physically present robot. Our results showed little difference between the virtual, remote, and physical experiments. These experiments reinforced our original finding that a two armed robot provided the best emergency evacuation guidance.

In addition to conveying directions, an emergency guidance robot must also localize itself on a map of the environment, move past or around static obstacles, and be generally capable of moving to a guidance point in order to direct evacuees. Additional perceptual capabilities, such as recognizing people, identifying the direction of their movement, counting people, and recognizing crowded exits would be beneficial but are currently in the early stages of development.

### Simulation Versus Real-World Experiments

One important and challenging aspect of robot-guided emergency evacuation research is the need to create as realistic an emergency as possible. A large body of evidence suggests that emergencies activate fight-or-flight responses which strongly influence how evacuees make decisions (Klein et al., 1986; Jansen et al., 1995). The fight-or-flight responses are only triggered when the subject believes that they may be in danger. Yet generating fictitious, yet convincing, emergencies is difficult and must only be undertaken with care. In real-world experiments sham emergencies could put the subject at risk if they panic. On the other hand, if the emergency is not convincing then the validity of the data is uncertain. Moreover, for real-world experiments, creating a convincing sham emergency is difficult given that subjects know that they are participating in an experiment. In the past we have, for example, used smoke machines to fill rooms and hallways with smoke in order to make the emergency convincing (Robinette et al., 2016c). But creating convincing sham emergencies that do not actually endanger the participant and are acceptable to an institutional review board is challenging. Furthermore, word that the emergency is a sham may spread quickly among potential subjects if the experiment is conducted at a university. Thus experiments must be conducted quickly, over only a few days and nights, if possible.

Simulation experiments offer the possibility of not only testing out a wider variety of experimental conditions, but also much easier methods for generating sham emergencies. Simulation effects such as sirens, flashing-lights, explosions, smoke, and fire are all available and easily incorporated into a simulation environments such as Unity. Moreover, services such as Amazon Mechanical Turk provide a very large and diverse pool of potential subjects. The problem with simulation experiments, however, is creating convincing and engaging sham emergencies and the resulting validity of the subject’s responses. Our lab has conducted a large number of emergency evacuation simulation studies (Robinette et al., 2016b; Robinette et al., 2017). We have also conducted physical experiments attempting to confirm (or refute) these prior simulation studies with mixed results. There is much more work that needs to be done in this area. We are now attempting to use virtual reality as a method to more realistically engage subjects in simulate emergencies. Our hope is to find an ideal middle ground that will allow us to test a wide range of variables in a manner that results in ecologically valid responses. If we can achieve such a balance, then real-world testing of the most promising variables can commence. We believe that the HRI field would benefit from the development of a well-honed process that begins with large scale simulation (or virtual reality) based testing of social phenomena but then leads to a small number of ecologically valid experiments of the most promising factors and hypotheses.

## Evacuation as an Paradigm for Human-Robot Interaction

**FIGURE 7:**
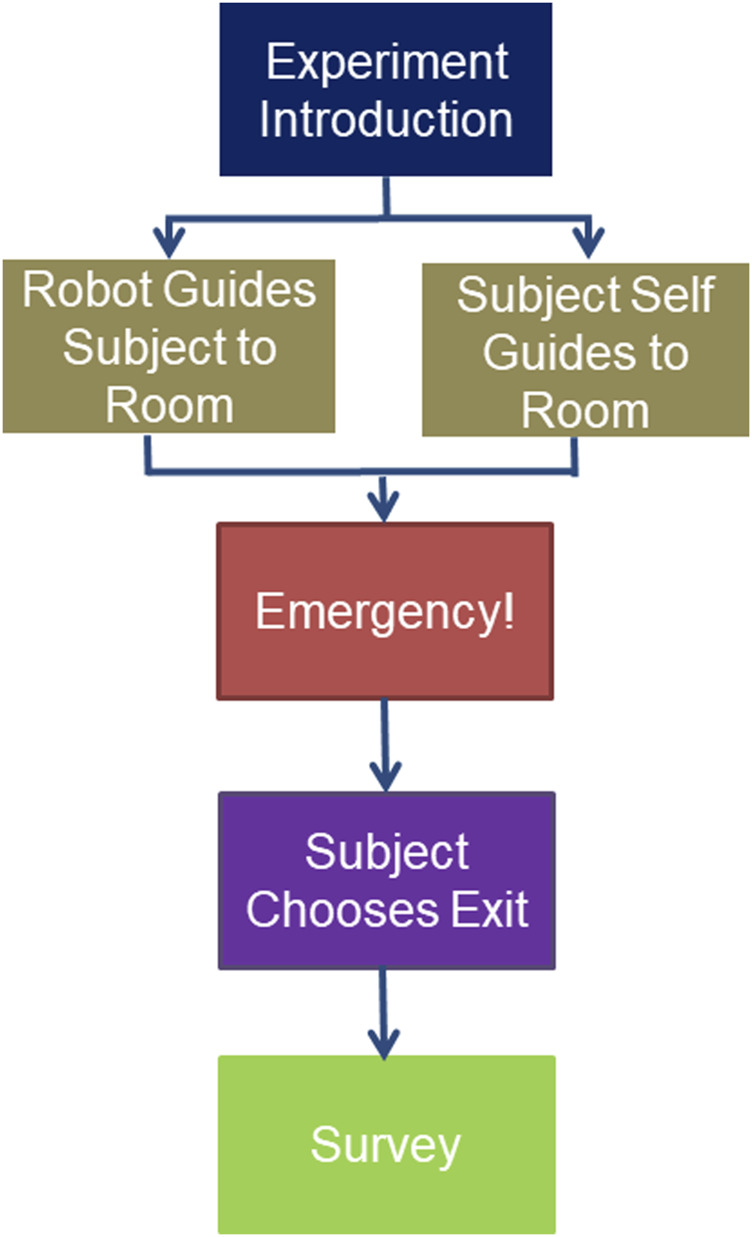
The general procedure for robot-guided emergency evacuation experiments. The subject is introduced to the robot. The robot guides the subject to a meeting room or the subject self-guides to a meeting room. An emergency occurs. The robot approaches subject asking the subject if they would like to follow the robot to an exit. After reaching (or not reaching) the exit the subject completes a survey.

Considering the different approaches to robot-guided emergency evacuation described above, there are several experimental designs that can be used for human subject testing. These different experimental designs attempt to measure whether or not human subjects will follow a robot’s guidance to an exit after an unexpected emergency has occurred and contribute to the design of robots that promote trust calibration (Wagner et al., 2018). Understanding how human subjects respond to the guidance instructions of an evacuation robot is critical for the design and application of useful emergency evacuation robots. Our robot-guided evacuation experiments typically introduce the human subject to the robot, ask the subject to complete a nominal task, an emergency occurs, and the robot offers to guide the person to a safe exit (Figure 7). The percentage of people that follow the robot represents a metric of not only the robot’s usefulness, but also of the person’s trust in the robot. In a real-world application, when an emergency occurs the robot will travel to guidance points to guide evacuees to safety.

### Metrics for Measuring Evacuation Success

One advantage of using robot-guided evacuation as a paradigm for studying human-robot interaction is that evacuation has very intuitive and well-defined metrics for success (Gershon et al., 2007; Gershon et al., 2012). Generally speaking, the success of an evacuation is measured by two criteria: the percent of people evacuated and the rate of evacuation. The percent of people evacuated is often not known until after the emergency has ended and casualty rates are known or estimated. The rate of evacuation is generally measured in terms the time required to evacuate a percentage of people. For robot-guided emergency evacuation these two metrics offer a means to evaluate and compare different evacuation approaches, robots, control algorithms, and methods of communication. Of course, many additional factors, such as the characteristics of the evacuees and the type of emergency, also influence these metrics. Nevertheless, simply having metrics for quantifying the performance of a human-robot interaction paradigm helps to ground the problem and make it more tractable.

The connection between evacuation rate and the robot’s guidance assumes that the presence of the robot will decrease the time that it takes the evacuee to exit. This can occur in several ways. The most obvious is if the robot guides the evacuee to a closer exit than they would have other traveled to. A less obvious, but more realistic way in which robots can decrease evacuation time is for the robot to prompt or pressure the person to evacuate. It is often noted in the evacuation research that one of the biggest challenges associated with an emergency is getting people to evacuate in the first place. As noted earlier, 6 h after an explosion occurred under the world trade center people were still found at their desks (Fahy RF, 1995). A robot might compel straggling evacuees to move to an exit by directing individualized messages at the stragglers. The development of evacuation choke points is a significant risk during some types of emergencies (Robinette et al., 2012). Hence, one final way that a robot could be able to increase the evacuation rate is by attempting to redirect evacuees away from choke points and toward less crowded exits.

If one assumes that the robot’s evacuation guidance will result in an evacuation rate increase, then a critical metric is evacuee compliance with the robot’s guidance. In other words, measuring how often and for how long people will follow the robot. Intuitively, even if an evacuation robot is excellent at its job, it makes no difference if few people follow it. Evacuee perception of the robot, measured by questionnaires such as the Godspeed survey, can also be useful for gauging the robot’s effectiveness.

### Independent Variables: The Environment, the Robot, the Evacuee

**FIGURE 8:**
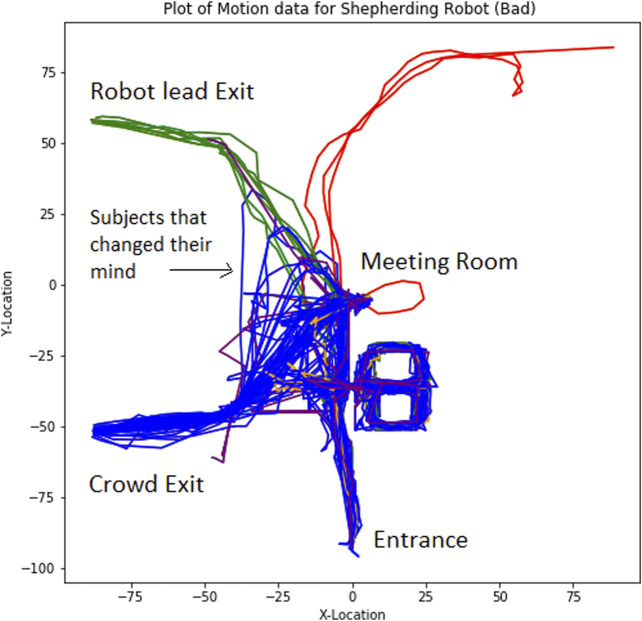
A map of subject movements (60 subjects are depicted). The blue lines depict movement following a crowd of evacuees. The green line depicts subjects that followed the robot. Notice some blue lines appear to initially move toward the robot exit, before following the crowd.

Given the above metrics and the described experimental setup, we now consider the different types of variables that can be examined. We broadly categorize these variables as environmental, robot-related, and evacuee-related. Environmental variables include the type of emergency faced, the level of uncertainty generated by that emergency, the familiarity of the person with the environment, the presence or absence of family members or a social group, and other environment-related issues. Simulation experiments allow one to broadly explore many different aspects of the environment. The design of the robot may also influence a human subject’s decision to follow the robot. Robot-related variables may include the robot’s form factor, mobility, ability to attract an evacuee’s attention, and its ability to interact with evacuees including answering questions. The robot’s ability to explain its directions or the need for evacuation may be an important determinate of the person’s decision to follow the robot. Similarly, the robot’s mannerisms and behavior must appear authoritative in order to promote compliance and induce evacuees to follow its directions. Finally, characteristics of the evacuee(s) will also shape the decision to follow the robot. Age, mobility, the presence of disabilities, and occupation, may influence the evacuee’s following behavior. Moreover, one’s personal experiences, including experiences with robots, can impact the decision or hesitancy to follow a robot’s guidance during an emergency. Importantly, although we are describing the decision to follow as an all-or-nothing choice, in practice, evacuees may initially follow the robot and then change their mind. Recording the evacuee’s movements (Figure 8) provides insight into the decision making process and, we have found, often conflicts with what people say about their own behavior (Nayyar and Wagner, 2019). Experimentally, we can attempt to isolate these variables in order to evaluate the influence each one has on the subject. In practice, because of the number of permutations of these variables, this is practical only in simulation.

## Design Aspirations for Evacuation Robotics

Our research and reflection on the topic of robot-guided emergency evacuation has resulted in the development of several design aspirations for evacuation robotics. These aspirations are meant to serve as an initial set of guiding principles, open to future refinement if necessary, for researchers interested in the topic of robot-guided emergency evacuation. These are aspirations in the sense that they are meant to encompass somewhat abstract design and ethics goals for these types of systems. For example, our hope is that researchers will aspire to design evacuation robots that can communicate understandably with as diverse a population as possible. Importantly, it is hoped that these principles will ensure that the development of these technologies will positively impact future societies.

• Principle 1: Do no harm. An evacuation robot must not hinder an evacuation. It must not mistakenly direct evacuees toward danger, delay evacuation by blocking passageways or exits, or slow evacuation by drawing interest to itself. It is better to not have evacuation robots than to have evacuation robots that may increase the risk to the evacuee. Furthermore, robots should only be deployed in situations in which the “Do no harm” principle can be reasonably guaranteed. The primary purpose for this principle is to prevent the premature deployment and justification for evacuation robots. Evacuation robots should only be deployed if the developer has shown that the system will do no harm. The use of shoddy or untested evacuation robots on the basis that they are better than nothing at all should be avoided.
• Principle 2: Communicate understandably with as diverse a population as possible. Evacuation robots must be designed to communicate with a diverse population of evacuees. Explicit or implicit limitations on the robot’s ability to communicate could inadvertently increase the survival rate of some evacuees over others. For example, limiting evacuation directions to English could disadvantage non-English speakers attempting to evacuate. Hence, an evacuation robot’s method of communication should not be designed for a narrow or predetermined population. Evacuation directions should be understandable, within reason, regardless of age or native language. Moreover, we encourage the use of gestures, lights, signs on the robot and audio messages in order to promote the robot’s ability to communicate with individuals with disabilities. Extensive testing should be conducted with as diverse a population of subjects as possible to measure whether the robot’s guidance directions are understandable (Robinette et al., 2016a). This evaluation should include reasonable environmental conditions, such as recognizing commands from a distance, or while being distracted.
• Principle 3. Be authoritative. An evacuation robot should act and be seen as an authority figure during an emergency. Acting as an authority figure may be necessary to generate compliance from evacuees. Command presence is defined as presenting one’s self as someone in authority (Mitchell and Von Zoller, 2019). Robots will need to either imitate human command presence or develop a set of behaviors that generate a type of robot command presence. Lights, behaviors, and mannerisms can be used to establish the robot as an authority figure during an emergency. Police or emergency style beacon lights, forceful behaviors and gestures, or authoritative commandments can be used by the robot to improve evacuee compliance. Childish or overly commercial designs should be avoided.
• Principle 4. Attract attention, but also keep interactions minimal. An evacuation robot should attract an evacuee’s attention in order to provide guidance to an exit, but must also keep interactions short and focused on directing the evacuee to the exit. Evacuees may be distracted by the sights and sounds of the emergency, alarms, and movement of the people around them. Capturing the evacuee’s attention in such a situation can be difficult. An evacuation robot should use movements, lights, and sounds to attract evacuee attention. Once the robot has captured an evacuee’s attention it must communicate directions to the exit quickly and precisely. It should otherwise minimize interactions with evacuees. In spite of the emergency, the evacuee may slow their evacuation to engage or marvel at the robot. The robot should not engage in question and answer sessions, lengthy explanations, or allow the person to gape at the robot. The robot must not encourage evacuees to preoccupy themselves with the robot during the emergency. This can be challenging, especially if the alarm is not deemed credible or if the robot is a novelty. Hence, balancing the robot’s ability to attract attention and yet keep interactions minimal is an important design goal.
• Principle 5. When the situation demands it, evacuate as many people as possible, as quickly as possible. Saving as many lives as possible is an evacuation robot’s ultimate goal. Different emergencies, however, demand different approaches to obtaining this goal. During a fire the robot should quickly guide evacuees to an exit. The performance of an evacuation robot during a fire is based on its ability to quickly lead as many people as possible to an unobstructed exit. During an active school shooting, on the other hand, the robot should guide students and staff to shelter in place. In this case the robot’s performance may need to be evaluated in terms of its ability to relay information about the evolving situation to the students and staff. A variety of different factors, such as characteristics of the evacuees, the environment, or the emergency, can impact the robot’s performance. Nevertheless, the design of the robot must always be centered on saving as many lives as possible.

## Ethics of Robot Evacuation

The possibility of creating an emergency evacuation robot raises a number of important ethical considerations. Robot-guided emergency evacuation generates both robot ethics and machine ethics questions. Robot ethics examines the ethical problems that arise when using robots (Lin et al., 2014). For example, recognizing and ensuring that an evacuation robot does not preferentially select some evacuees over others is a robot ethics question. Machine ethics, on the other hand, explores how to create robots that act ethically (Moor, 2006). Developing algorithms that allow robots to recognize and use explanations to prevent overtrust is an example of a machine ethics facet of this work.

The development of an evacuation robot might change the nature of evacuation itself. Currently, once an alarm is sounded evacuees decide for themselves how to respond. For many people, the typical response is to do nothing and assume that the alarm is a false alarm (Winerman, 2004). An evacuation robot might use a variety of different means to dissuade people from remaining in a building. As mentioned in the previous section, we contend that acting as an authority figure to demand that the people leave is ethically acceptable based on the assumption that the robot is trying to save lives. On the other hand, a robot that threatens people that refuse to evacuee is likely unethical. Although different situations and evacuees may require different persuasive approaches, a robot that threatens or menaces evacuees in order to gain compliance is likely beyond that bounds of acceptable behavior. The use of deception to gain compliance may be ethical in some situations and unethical in others. First responders, for example, may omit information, such as the demise of a loved one, if they believe that such information will distract or dissuade an evacuee from leaving. It may be acceptable and necessary for future versions of evacuation robots to similarly omit such information in similar situations. On the other hand, the general use of deception, exaggeration, or lies in order generate compliance is likely unethical.

Futuristic versions of emergency evacuation robots could present additional ethical considerations, especially if these robots are designed to make decisions about who to evacuate first. Yet, if we assume that the robot has the capability to move an injured person to safety, we contend that it then becomes reasonable for the robot to decide who to move first. These types of triage decisions are challenging even for humans (Grimaldi, 2007; Holt, 2008). Cultural and experience-based beliefs can play a role. If future evacuation robots are developed with the ability to move people to safety it will be important for the scientific and broader community to discuss and develop rules for whom to save first.

The robot-guided emergency evacuation problem also offers a venue for the development of machine ethics related technology. In particular, developing technology that allows a robot to explain to people why they should evacuate, observe their reaction, and then, if needed, reformulate the explanation is important for some approaches to robot-guided emergency evacuation. Additional, developing methods that allow authorized first responders and medical personnel to observe an evolving emergency while also protecting the privacy and medical information of the observed will also require the development of specialized technology.

## Conclusion

This paper has presented a conceptual outline for the problem of robot-guided emergency evacuation. Our purpose is to introduce this problem as well as the technological and interactive challenges that must be solved in order to create robotic evacuation solutions. We believe that the investigation of this problem offers a novel and important opportunity to investigate human-robot interaction in situations in which the human is reacting in an emotion inducing, stressful situation. We feel that it is important explore how people interact with robots during trying situations. The results from research on this problem may lend insight into understanding how a robot should interact with a frightened child or a terminally ill patient. Further, if successful, this research may also one day save lives during real evacuations.

Although the presence of an evacuation robot might alleviate some of the challenges of emergency evacuation, it is possible, however, that the use of robots could cause other issues. For example, our research has demonstrated that evacuees tend to overtrust an evacuation robot (Robinette et al., 2016c). Hence, they may follow a broken or lost robot, putting themselves at greater risk. Further, evacuees may simply wait for the robot or some sign of the robot before they begin evacuating, thus increasing the time required to evacuate and reducing the evacuation rate. People may also intentionally block, mob or prevent the robot from moving, even during an evacuation. This type of behavior has been witnessed in children in non-emergency settings (Nomura et al., 2016). Similarly, first responders may come to overtrust the ability of evacuation robots to lead people to safety, reducing their sense of urgency to assist. Moreover, the information provided by the robot may focus on some aspects of the emergency, drawing the attention of first responders away from other risks. For example, if the robot’s camera searches for and focuses on injured humans then it may draw the attention of first responders away from other dangers, such as a fire. In general, experimental evaluation and rigorous testing should highlight and help prevent most of these concerns from occurring in fielded systems.

A roadmap for robot-guided emergency evacuation would likely begin with simple traffic-cop style robots that move to nearby locations during an emergency. These robots could also serve some other purpose, typically cleaning hallway floors, for example, but spring into action once an alarm is sounded. Additional features, such as allowing first responders to take over control of the robots, can be added gradually with significant testing. As methods for perception and more capable, cost effective robots become available, robots that shepherd evacuees to exits can be implemented. Eventually we hope the field will work toward systems that become autonomous yet active partners in the rescue of victims during an emergency.

We hope and believe that one day robots will save lives during emergencies by thoughtfully and carefully leading people to safety. Such an application could contribute the peace of mind necessary to focus on learning, entertainment and one’s long-term health.
